# Important players in carcinogenesis as potential targets in cancer therapy: an update

**DOI:** 10.18632/oncotarget.27689

**Published:** 2020-08-11

**Authors:** Justyna Magdalena Hermanowicz, Iwona Kwiatkowska, Dariusz Pawlak

**Affiliations:** ^1^Department of Pharmacodynamics, Medical University of Bialystok, Mickiewicza, Bialystok, Poland; ^2^Department of Clinical Pharmacy, Medical University of Bialystok, Mickiewicza, Bialystok, Poland

**Keywords:** modulators of carcinogenesis, acetylcholine, peroxisome proliferator-activated receptors, aquaporins

## Abstract

The development of cancer is a problem that has accompanied mankind for years. The growing number of cases, emerging drug resistance, and the need to reduce the serious side effects of pharmacotherapy are forcing scientists to better understand the complex mechanisms responsible for the initiation, promotion, and progression of the disease. This paper discusses the modulation of the particular stages of carcinogenesis by selected physiological factors, including: acetylcholine (ACh), peroxisome proliferator-activated receptors (PPAR), fatty acid-binding proteins (FABPs), Bruton’s tyrosine kinase (Btk), aquaporins (AQPs), insulin-like growth factor-2 (IGF-2), and exosomes. Understanding their role may contribute to the development of more effective and safer therapies based on new binding sites.

## INTRODUCTION

**Figure 1:**
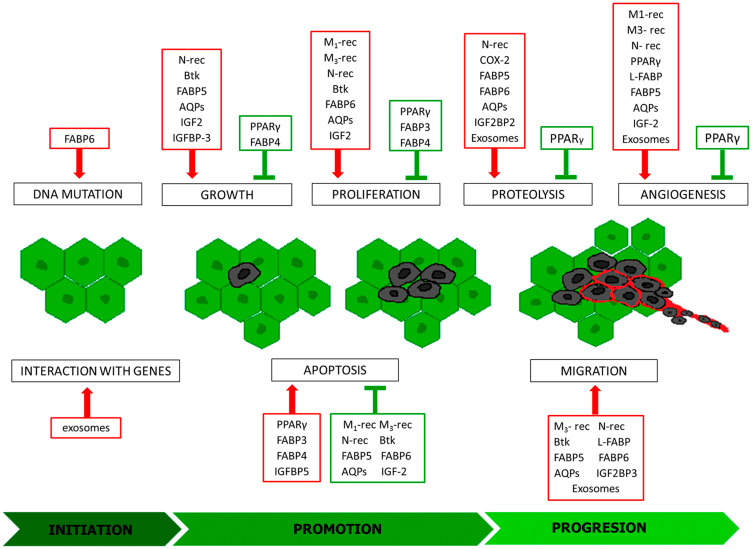
Modulators of individual stages of carcinogenesis. Inducers: red frames, inhibitors green frames; M1-rec: muscarinic receptor 1; M3-rec: muscarinic receptor 3; N-rec: nicotinic acetylcholine receptor; COX-2: cyclooxygenase-2; PPARγ: peroxisome proliferator-activated receptors gamma; Btk: Bruton’s tyrosine kinase; FABP 3-6: fatty acid-binding protein 3-6; L-FABP: L-type fatty acid-binding protein; AQPs: aquaporins; IGF2: insulin-like growth factor 2; IGFBP-3: insulin-like growth factor binding protein 3; IGFBP5: Insulin Like Growth Factor Binding Protein 5; IGF2BP2: Insulin Like Growth Factor-2 mRNA Binding Protein-2; IGF2BP3: Insulin Like Growth Factor-2 mRNA Binding Protein- 3.

The increasing prevalence of various types of cancers is a global phenomenon that has been occurring widely and affecting increasing numbers of people. In 2018, over 18 million new cases were reported and statistics show that every fifth man and every sixth woman is at risk for developing cancer [1]. Carcinogenesis is a multi-stage process that leads to cancer development. It includes three main stages, initiation, promotion and progression, and each is characterized by different processes. During the promotion stage the genetic material of cells is damaged and if not detected by repair systems before the next division, the change is transferred to newly formed cells. Under the control of growth factors, initiated cells may move on to the second stage of cancer formation–promotion. During that stage, mutated cells undergo multiple divisions, their growth is uninhibited as they become insensitive to apoptotic signals. As a result, the tumor increases its mass, but the cells remain within one organ. The formation of metastases occurs in the next phase–progression. Metalloproteinases secreted by tumor cells destroy the extracellular matrix. This allows them to enter the bloodstream and reach a new organ, at a considerable distance from the primary lesion, with the stream of flowing blood. The key step for the survival of migrating cells is adhesion to the attacked organ and the formation of new blood vessels, thanks to which they will receive the components necessary for development. Angiogenesis, the formation of new blood vessels, is the last element of carcinogenesis and is enhanced by tumor cell derived factors, metabolic changes, and a decrease in available oxygen. Each of these processes is controlled by different signal pathways via proteins that naturally occur in the body (Figure 1). Dysregulation of their expression, and thus activity, both to an excessive and insufficient degree, ensures the continuity of carcinogenesis and increases the chances cancer cells survive in a host organism. Understanding the role of individual substances and components of the body in the subsequent stages of neoplastic transformation provides a more complete picture of cancer pathogenesis. This allows to develop new recommendations, to improve the quality and safety of pharmacotherapy, which has a direct effect on an improvement in the cancer patients’ quality of life. It also provides the basis for research aimed at developing new compounds that are effective weapons in the fight against cancer. Therefore, there is no doubt that any actions that bring scientists closer to solving the problem of insufficient cancer therapy are sensible and much needed. The purpose of this paper was to discuss the role of selected physiological factors in the various stages of neoplastic transformation.

### Crucial mechanisms involved in carcinogenesis

The carcinogenic process is regulated by many molecular and cellular mechanisms. The diversity of signaling pathways exploited during cancer initiation, promotion and progression is immense. Many of them are common in different cancer types, but there are also clearly unique molecular and cellular signaling signatures specific for particular cancers [2]. Pathways such as Akt-PI3K, Ras/MAPK/ERK, Wnt/β-catenin, JAK-STAT occupy crucial roles in the regulation of cell proliferation, apoptosis, differentiation, angiogenesis, cell migration and invasion, immunological activities, and inflammation.

PI3K, phosphatidylinositol 3-kinase, is a family of enzymes that after activation by growth factors, cytokines, and hormones are involved in the conversion of PIP2 (phosphatidylinositol-4,5-bisphosphate) into PIP3 (phosphatidylinositol (3,4,5)-trisphosphate) [3]. The latter in turn takes part in recruiting Akt into the cell membrane, where it is phosphorylated and activates other agents, such as mTOR, NF-kB, Wnt or inhibits factors like Bad, p27 to enhance carcinogenesis. Dysregulation of this pathway occurs in endometrial [4], bladder [5] or gastric cancers [6]. The aforementioned PIP2 can be also transformed by phospholipase C into DAG and IP3, which then activate protein kinase C (PKC) [6]. One of the PKC substrates is Raf kinase, which is an element of the Ras/MAPK/ERK pathway, which is involved in the pathogenesis of ovary cancer [7] or non-small cell lung cancer [8]. Ras proteins activate Raf, which then phosphorylates kinases MEK1/2. Its final substrate is kinase ERK1/2, which after transportation into the nuclei regulates transcription factors, essential for DNA synthesis and cell cycle progression. They condition cell growth, proliferation and viability [9]. Because of that, targeting those two cascades as an anticancer therapy seems to be beneficial. However, it is important to notice that the existing crosstalk between both of the pathways can impede treatment, and inhibition of one can strengthen the activity of the other. Another component that is also connected with the MAPK family is a signaling pathway conducted by p38MAPKs. This subfamily contains four kinases, which are activated mainly by inflammatory cytokines and stress factors. p38MAPKs control many aspects of cell physiology such as cell cycle regulation, differentiation or skeleton remodelling. Additional p38MPAKs can act as tumor promotors by enhancing metalloproteinase and VEGF expression [10].

Wnt/β-catenin signaling plays essential roles in caricinogenesis. Activation of canonical Wnt signaling results in the inhibition of GSK-3β kinase, dissociation of β-catenin proteins and its transfer to the nucleus where it regulates multiple downstream genes like c-Myc and cyclin D [11]. This axis was found to be associated with several oncogenic events including tumor cell proliferation, migration, epithelial–mesenchymal transition and invasion. An abnormally active Wnt/B-catenin pathway is observed, among others in gastric [12], colon [13], breast [14], or adrenocortical cancer [15]. Another signaling pathway involved in the pathogenesis of many cancer types is that induced by the Jak kinases family. It includes four kinases, which after activation impact of the STAT molecules causing their translocation into the nuclei [16]. STAT, a family that gathers seven forms of proteins, is associated with cancer cell development, progression, metastasis, survival and resistance to treatment [17]. STAT3 and STAT5 factors seem to be the most important agents in light of cancer progression. They have an impact on p53 protein, which leads to a disruption in cycle control and apoptosis and induces cell proliferation by the increased c-Myc and cyclin D expression. By the induction of VEGF gene expression, they take part in enhanced angiogenesis and induction of genes such as survivin, Bcl-2, Bcl-XL conditioning overall survival of the tumor cells. Moreover, pSTAT3 acts to negatively regulate neutrophils, NK cells, effector T cells and dendritic cells, while positively regulating populations of MDSCs (myeloid-derived suppressor cells) and regulatory T cell leads to a highly immunosuppressive TME (tumor microenvironment) [18].

Multiple endogenous factors are involved in carcinogenesis, which affects particular stages of carcinogenesis in various mechanisms. Some of them interact directly with the DNA, intracellular signalling molecules, others by specific cellular receptors. Among them are well known growth factors (EGF, TGF-α and TGF-β, FGF) or reactive endogenously generated oxygen molecules as well as less often described agents like ACh, PPAR, FABPs, Btk, AQPs, IGF-2, or exosomes. The latter have only recently gained in importance and are more widely studied and discussed therefore are considered in this article.

### Aacetylcholine (Ach) and its receptors

**Figure 2:**
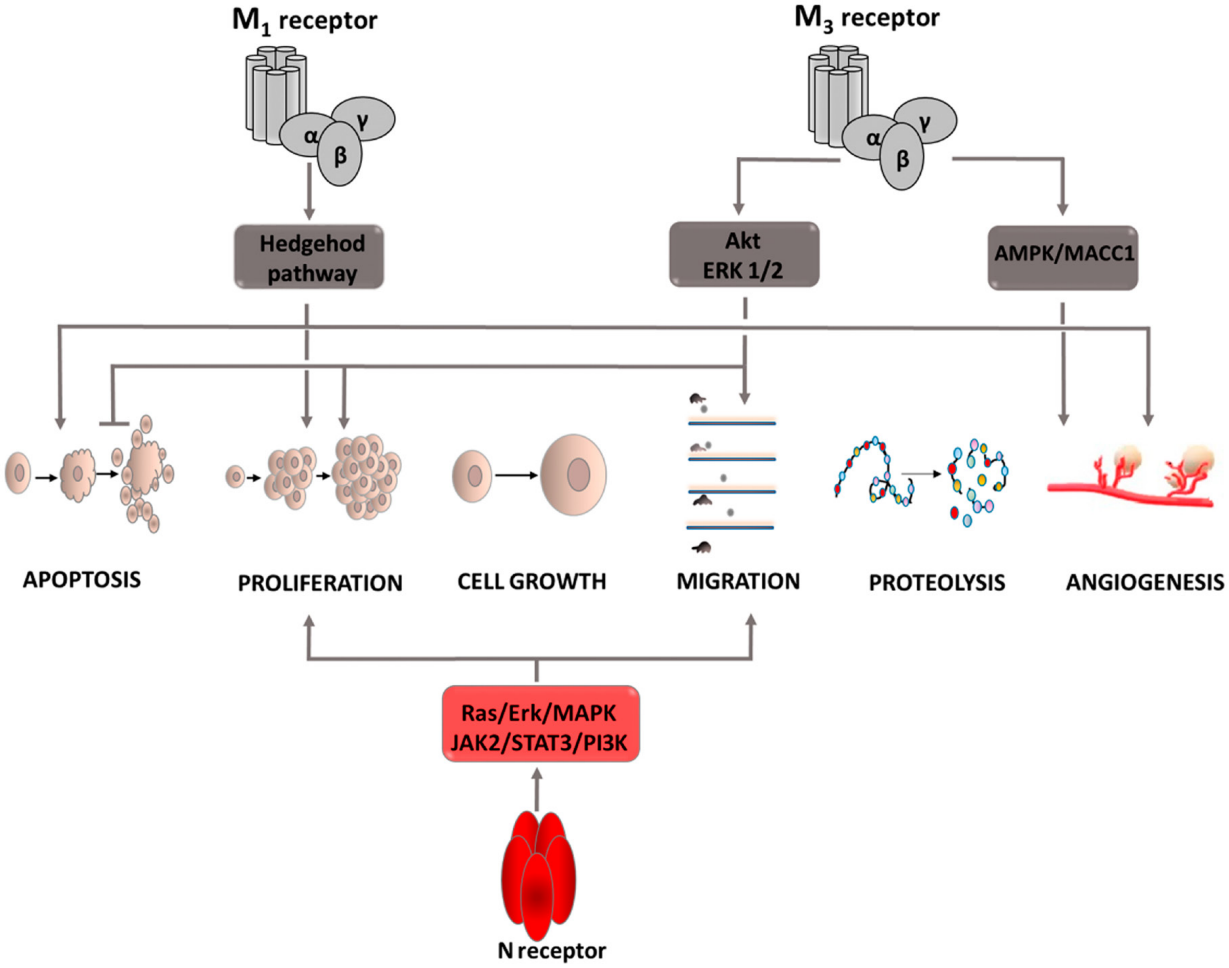
Carcinogenic effect of acetylcholine (Ach). Intracellular signaling pathways activated by ACh via M1 and M3 receptors. Induction of process is illustrated by arrows, inhibition by horizontal lines. M1: muscarinic receptor 1; M3: muscarinic receptor 3; N receptor: nicotinic acetylcholine receptor; Akt: protein kinase B; ERK: extracellular signal-regulated kinase; JAK2: Janus kinase 2; PI3K: phosphoinositide 3-*kinase*; STAT3: signal transducer and activator of transcription proteins; MAPK: mitogen-activated protein kinase.

One of the factors involved in the carcinogenesis process is acetylcholine and its receptors, both muscarinic and nicotinic. Their expression has been demonstrated in numerous types of cancer cells [19–21]. It has also been proven that these cells contain acetylcholinesterase (AChE), an enzyme that enables cells to produce ACh in the absence of agonists from the external environment. Cancer cells also have the ability to produce autoantibodies that stimulate one of the muscarinic receptor subtypes, M3. These facts prompted more extensive research to explain the exact role of ACh and its receptors in the carcinogenesis process (Figure 2). Muscarinic receptors can be divided into five subtypes, each of which is involved in tumor development through a different mechanism. The role of muscarinic M3 receptor is the most widely described subtype. It is involved in the pathogenesis of lung, colon, gastric, and breast cancer. In the course of colon cancer, it has been observed that stimulation of the M3 receptor causes phosphorylation of the Akt and ERK1/2 kinases, which are factors that through the ability to regulate the activity of pro- and anti-apoptotic proteins affect the intensification of cell proliferation, survival and motility. In addition, co-expression of the M3 receptor and endothelial growth factor receptor (EGFR) as well as EGFR transactivation after M3 receptor stimulation has been demonstrated [22, 23]. Moreover, the administration of EGFR inhibitors inhibits acetylcholine-induced ERK1/2 kinase phosphorylation, which is reflected in a significant decrease in colon cancer cell proliferation. This indicates that acetylcholine is involved in the formation of colon cancer, but for its full action it is necessary to activate the EGFR receptor. Thus indicating that enriching colon cancer treatment with EGFR inhibitors may improve patient outcomes. Acetylcholine is also involved in the formation of vascular-like structures in the vicinity of a developing tumor, guaranteeing the supply of essential nutrients to the proliferating cancer cells. This is called vascular mimicry. The basis of this process is the acetylcholine-dependent regulation of metastasis-associated in colon cancer-1 (MACC1) oncogene expression via the M3R/AMPK/MACC1 signaling pathway [24]. High activity of this oncogene is associated with an increase in cell invasiveness, intensified epithelial–mesenchymal transition, more frequent metastases, and hence worse treatment prognosis [25–27]. The first subtype of the M receptor (M1) is also involved in the tumor development process. It activates the hedgehog pathway [19, 28] whose physiological role is to regulate the transcription factors affecting the oncogenic activity of Gli (zinc finger transcription factors) [29, 30]. Excessive stimulation causes acceleration of carcinogenesis, intensification of proliferation, growth and survival of cancer cells as well as angiogenesis by affecting the expression of genes such as cyclin D, anti-apoptotic Bcl-2, or VEGF. Furthermore, stimulation of this pathway promotes the creation of a microenvironment conducive to tumor growth through increased expression of extracellular matrix components [31]. Nicotinic acetylcholine receptors are part of the ligand-gated ion channels, and are created from two types of subunits, α and β. Receptors that contain an alpha7 or alpha9 subunit in their structure are characterized by a high degree of permeability to calcium ions, thanks to which they are involved in the course of numerous processes based on signaling involving secondary messengers. Thus, after activating N receptors, Ca^2+^ inflows into the cell, causing the secretion of growth factors exerting paracrine action on neighboring cells. Simultaneously, apoptosis is inhibited enabling further proliferation. Studies conducted on a lung cancer cell line after exposure to cigarette smoke have shown that nicotine and nitrosamines, which have a higher affinity for nicotinic acetylcholine receptors (nAChRs) than nicotine, bind to the alpha7 receptors activating the Ras/ERK/MAPK and the JAK2/STAT3/PI3K signaling pathways [32, 33]. The effect of this interaction is increased proliferation and migration of cancer cells, and thus facilitated metastasis creation. Moreover, by affecting B-adrenergic receptors, nicotine intensifies COX-2 expression, which through the p38 MAPK and the JNK pathways leads to an increase in VEGF production, a highly pro-angiogenic factor. In addition, the high activity of cyclooxygenase affects the inactivation of the PTEN protein [34, 35], whose biological role is silencing PI3K/Akt pathway signaling. This leads to cellular signal transition and induces effects that contribute to the enhanced growth and proliferation of cancer cells as well as inhibits the process of their death. Under the influence of the Akt kinase, proapoptotic transcription factors are inactivated, including kinase-9, mTOR kinase intensifying cell proliferation is activated, and VEGF is activated. This indicates the involvement of this pathway in processes that facilitate cancer cell survival. Another mechanism contributing to cancer development in which cyclooxygenase 2 is involved is the complex process involving the COX-2/PGE2/EP4 axis, as a result of which metalloproteinase 9 expression is stimulated [36–38]. MMP-9 causes proteolytic activation of TGF-β, which begins the induction of epithelial-mesenchymal transformation (EMT). During this transition, the cells acquire features that increase their invasiveness. This leads to epithelial cell phenotype changes, the connections between adjacent cells and between cells and the basement membrane are loosened, the cytoskeleton is rearranged, cells lose contact inhibition, thus causing excessive proliferation. Furthermore, the microenvironment of the growing tumor also changes, promoting features that facilitate the movement of the cancer cells from the primary focus to other regions of the body. Through the EMT process, cells acquire greater migration capacity, and thus their invasiveness increases. In this cellular process, MMP-9 also modulates the activity of integrins and other molecules that ensure cell adhesion [39], and secrete soluble factors into the bloodstream that facilitate the implantation of migrating cells in organs distal to the primary tumor focus [40]. Thanks to these processes, COX-2 enables the formation of cancer metastases.

### PPAR: peroxisome proliferator-activated receptors

**Figure 3:**
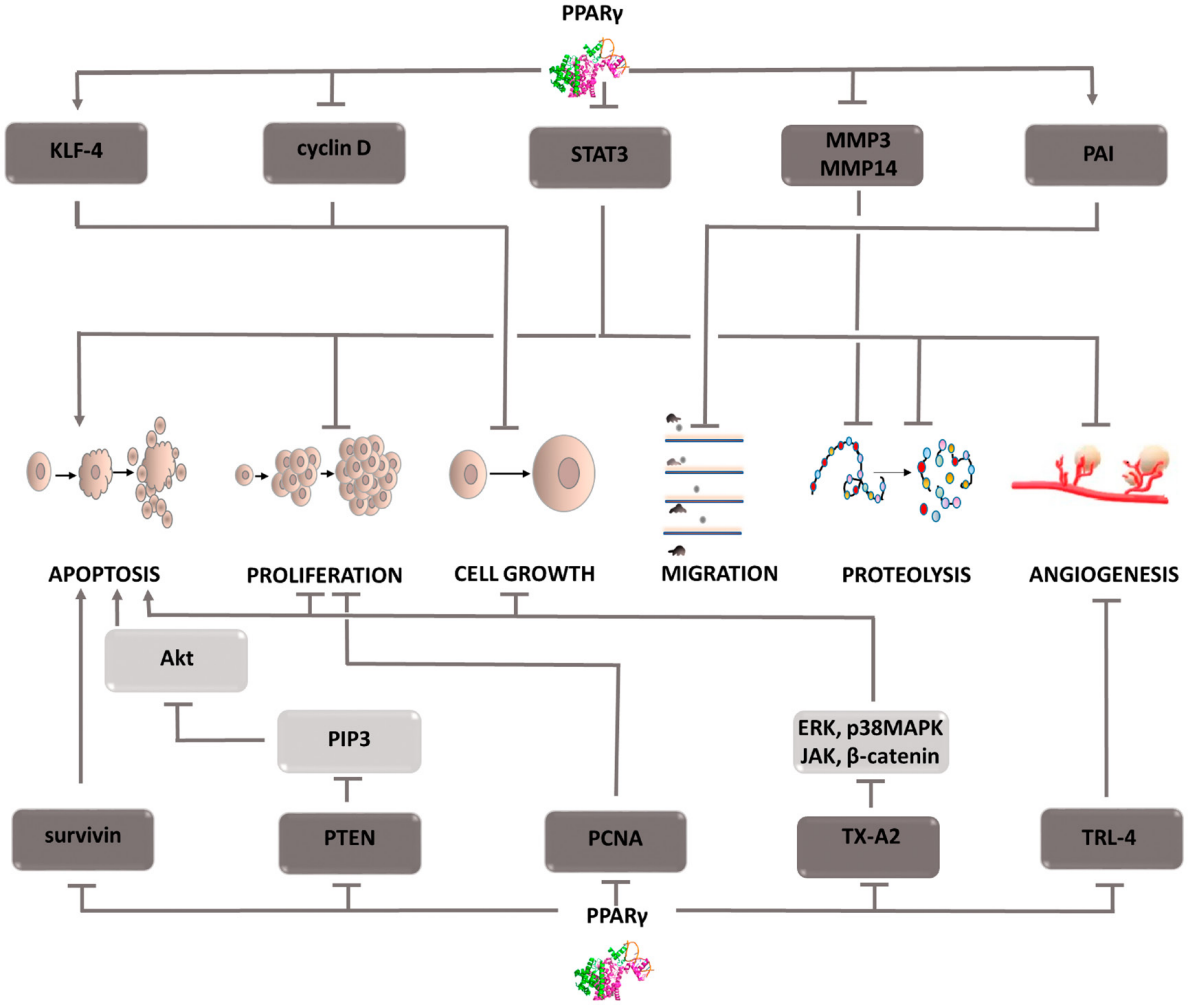
Carcinogenic effect of peroxisome proliferator-activated receptors (PPAR-γ). The induction of the process is illustrated by arrows, the inhibition by the horizontal line. PTEN: phosphatase and tensin homolog deleted on chromosome ten; PIP3: phosphatidylinositol (3,4,5)-trisphosphate; Akt: protein kinase B; PCNA: proliferating cell nuclear antigen; TX-A2: thromboxane A2; ERK: extracellular signal-regulated kinases; p38MAPK: P38 mitogen-activated protein kinases; JAK: Janus-activated kinases; KLF-4: Kruppel-like factor 4; STAT3: Signal transducer and activator of transcription 3; MMP3,14: metalloproteinase 3 or 14; PAI: Plasminogen activator inhibitor; TRL-4: toll-like receptor 4; MAPK: mitogen-activated protein kinase.

The family of peroxisome proliferator-activated receptors (PPARs) includes 3 isoforms of these proteins that function as transcription factors. Each type of steroid receptor differs in its location and affinity for ligands [41]. The best known is the function of the PPARγ form. Agonists of this receptor are a therapeutic option in the treatment of patients with diabetes. Current research provides evidence that stimulation of PPARγ receptors inhibits cell proliferation, vessel formation, and induces apoptosis (Figure 3) [42, 43]. These properties make PPARg agonists attractive candidates for anti-cancer drugs. To achieve full transcriptional activity, heterodimerization of PPARg with the retinoid X receptor is necessary [44, 45]. This interaction enables the promoter to bind with the target gene and regulate its expression. An example of a gene against which PPARg acts as a repressor is the vascular endothelial growth factor (VEGF) gene [46]. Thus, angiogenesis is inhibited, and this mechanism has been demonstrated in studies on human hepatocellular carcinoma (HCC) cells [47]. For this cancer, PPARg is also involved in inhibiting cell growth and attenuating cell migration and invasiveness. By upregulating KLF-4 (Krüppel-like factor 4) expression and inhibiting cyclin D1 expression, the cell cycle is inhibited. Furthermore, PPARg negatively affects the expression of transcription factor STAT3, thereby inhibiting proliferation, survival and metastasis. Lowering STAT3 levels also negatively affects the angiogenesis process (by inhibiting VEGF) depriving cancer cells of nutrients. PPARg activation in HHC cells also impairs their metastatic ability [48]. This is associated with the inhibition of the expression of metalloproteinases, MMP9 and MMP13, the increased expression of their inhibitor and E-cadherin, a protein conditioning cell adhesion. In addition, PPARg increases plasminogen activator inhibitor expression, thus inhibiting the breakdown of the extracellular matrix and basement membrane proteins, which also reduces cancer cell invasiveness. PPARg has also been shown to inhibit esophageal cancer [49]. The mechanism responsible for suppressing this tumor is MAPK signaling pathway inhibition. This process requires TLR4 (Toll-like receptor 4) inhibition, which allows PPARg agonists to be administered. TLR4 is a pro-carcinogenic protein and affects, among others, changes in the tumor microenvironment and the severity of angiogenesis. Therefore, in addition to the indirect effect on the MAPK cascade, its inhibition is an important element of tumor suppression. Studies on esophageal cancer cells indicate that PPARg stimulation leads to a decrease in PCNA (proliferating cell nuclear antigen factor) expression, which indicates inhibition of DNA replication [49]. In addition, PPARg activation intensifies the process of apoptosis, as evidenced by an increase in the expression of the active form of caspase 3 and the Bax gene, and a decrease in Bcl-2 expression. In non-small-cell lung cancer, PPARg stimulation inhibits the neoplastic process by regulating PTEN protein expression [50]. This protein is a tumor suppressor and its activation causes the dephosphorylation and inactivation of the PIP3 second messenger. As a result, Akt kinase activity is inhibited leading to a decrease in NF-kB expression. Due to PPARg stimulation by eicosapentaenoic acid (EPA), a relationship is sought between PPARg and COX-2 activity that uses EPA as a substrate for prostanoid production. The combination of PPARg agonists with COX-2 inhibitors has demonstrated a synergistic tumor-suppressing effect in studies on non-small-cell lung cancer. Researchers indicate significantly reduced thromboxane TX-A2 synthesis using combination therapy compared with monotherapy as the main mechanism of this phenomenon [51]. A decrease in its concentration limits signaling in four pathways, ERK, p38 MAPK, JAK, and β-catenin, limiting tumor growth [52]. Furthermore, it affects the arrest of the cell cycle in the G2/M phase, preventing further cell division and contributing to the reduction of the expression of survivin, an anti-apoptotic protein [53]. The described mechanisms affect the induction of the apoptosis process and inhibit cell cycle progression, which limits cancer development. The above examples indicate that peroxisome proliferator-activated receptors play a significant role in inhibiting the development of various types of cancer, which is why agonist compounds can be considered potential anti-cancer drugs. However, further comprehensive and extensive research is needed to identify potential applications and treatment regimens.

### Btk: Bruton’s tyrosine kinase

**Figure 4:**
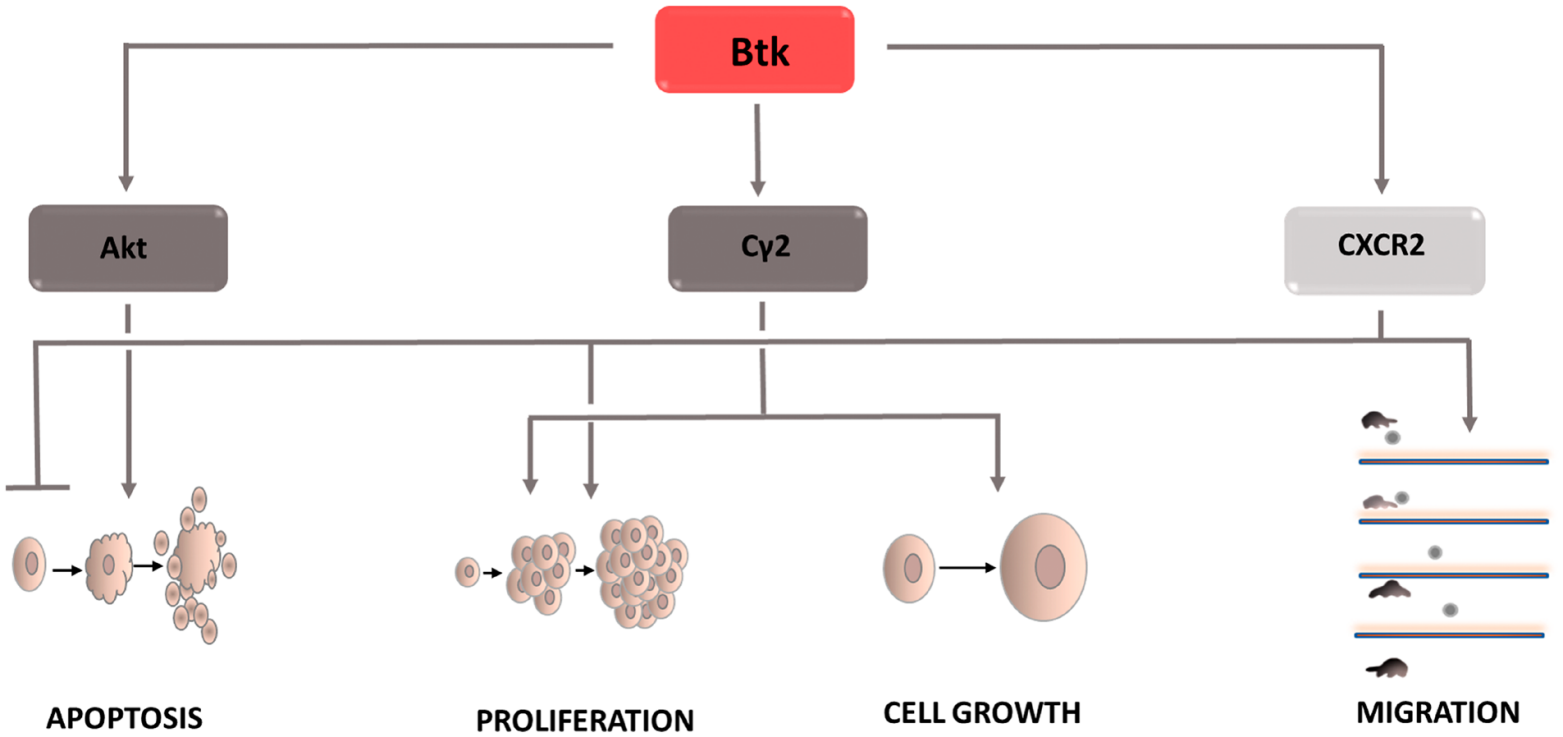
Carcinogenic effect of Bruton’s tyrosine kinase (Btk). The induction of the process is illustrated by arrows, the inhibiton by the horizontal line. Btk: Bruton’s tyrosine kinase; CXCR2: C-X-C chemokine receptor type 2; Cγ2: phospholipase Cγ2.

Bruton’s tyrosine kinase (Btk) also plays an important role in carcinogenesis (Figure 4). It is a key point in signal transduction from the BCR receptor leading to the activation of the NF-kB signaling pathway [54]. Btk affects phospholipase Cg2 [55], which after phosphorylation causes PIP2 hydrolysis. The result is IP3 and DAG. DAG activates protein kinase Cβ, which stimulates NF-kB pathway factors. Finally, genes conditioning cell survival are expressed. Btk also contributes to inhibiting the apoptosis process through interacting with the Akt kinase [56]. The signaling cascade includes active PI3K, which recruits Akt to the plasma membrane. Under the influence of Btk, its phosphorylation occurs, and thus its activation. Then, the active Akt returns to the cytoplasm and induces anti-apoptotic pathways dependent on NF-kB, among others. In addition, Btk, as a kinase belonging to the Tec family, affects intercellular signaling, causing changes in the tumor microenvironment. In most cancer cells, the developing microenvironment leads to the inhibition of immune processes, which results in protection, and protects defective cells from natural defense mechanisms. One of the most common mechanisms of this type is the effect of cancer cells on the increased differentiation of T lymphocytes towards Th2 cells. The biological role of these cells is the secretion of interleukins that start a humoral response dependent on B lymphocytes. Due to the increased differentiation of T lymphocytes in this direction, the number of formed Th1 lymphocytes directly destroying cells containing the abnormal genome decreases. The tumor microenvironment also interferes with normal dendritic cell activity. It inhibits their ability to migrate and to present the antigen to immune response cells. Some types of cancer show increased CXCL12 expression [57], which attracts immature dendritic cells and prevents their differentiation. In addition, this cytokine via the CXCR4 receptor causes a reorganization of the cytoskeleton [58] and increases cell motility [59], Tec family tyrosine kinases have also been shown to have the ability to activate CXCR4, which determines the growth, survival, and migration of tumor cells [60].

### FABPs: fatty acid-binding proteins

**Figure 5:**
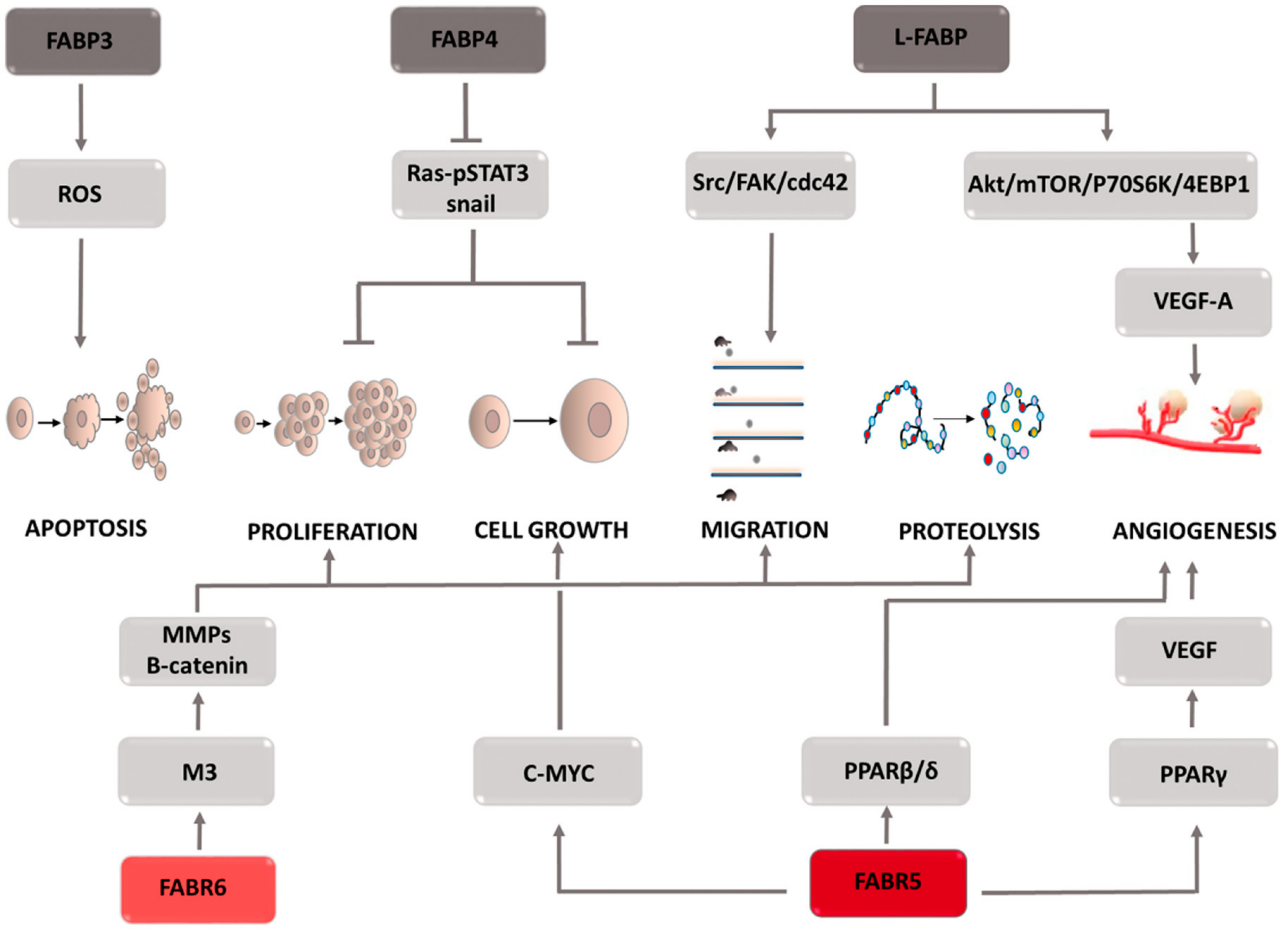
Carcinogenic effect of fatty acid binding proteins (FABPs). The induction of the process is illustrated by arrows, the inhibition by the horizontal line. STAT3: *signal transducer and activator of transcription 3*; Src: proto-oncogene tyrosine-protein *kinase Src*; FAK: focal adhesion kinase; cdc42: cell division control protein 42 Homolog; Akt: protein *kinase* B; mTOR: mammalian target of rapamycin; P70S6K: p70 ribosomal protein S6 kinase; 4EBP1: eukaryotic translation initiation factor 4E (eIF4E)-binding protein 1; VEGF-A: vascular endothelial growth factor-1; M3: muscarinic receptor 3; MMPs: *metalloproteinases*; PPARγ: peroxisome proliferator-activated receptors γ; PPARβ/δ: peroxisome proliferator-activated receptors β or δ.

The fatty acids supplied to the cell are involved in its metabolism. Due to their lipid nature, they are unable to enter the aqueous environment of the cellular cytoplasm. Solubilizing molecules, i.e., fatty acid-binding proteins (FABPs), enable their transport to various cellular structures and thus become involved in the processes taking place in the cells, including their growth, reproduction, and inflammatory processes. In cells characterized by intensive lipid metabolism, such as the liver, intestine, heart, brain, etc., high transporter expression is observed, which is associated with their physiological role in the cell. Depending on the type of cancer, both the overexpression and a decrease in FABPs can be seen. FABP isoforms are not specific to one type of cancer, and each plays a different role in the carcinogenesis process (Figure 5). In liver cancer, L-FABP was overexpressed and its high concentration was shown to correlate positively with VEGF-A [61–63]. The causes of this phenomenon are sought in the direct interaction between these two proteins, which in effect provokes the activation of the Src/FAK/cdc42 and the Akt/mTOR/P70S6K/4EBP1 signaling pathways. Activation of the Src/FAK/cdc42 pathway increases tumor cell migration, and L-FABP plays a key role in this process [64, 65]. Moreover, by stimulating the second mentioned signaling cascade, L-FABP enhances VEGF-A expression, facilitating angiogenesis. This process is regulated by HIF-1α, which additionally induces blood vessel formation [66]. Due to the high homology in their construction, FABP3 and FABP4 isoforms were assigned to the same protein subfamily. In addition to their physical features, it has been observed that the same factors, i.e., HIF1α, VEGF, increase their co-expression. The physiological role of both proteins is to inhibit excessive cell proliferation and increase apoptosis. Excessive FABP3 expression leads to an increase in reactive oxygen species and a decrease in the mitochondrial membrane potential [67], resulting in the opening of mitochondrial mega-channels, which play a key role in initiating apoptosis. FABP4 activates the apoptosis process by mediating the response to lipid-induced endoplasmic reticulum stress. Due to the biological functions of both proteins, their deficiency may be the cause of cancer progression. When examining the level of FABP3 expression in embryonic tumor cells, breast cancer cells [68], and FABP4 in breast, ovarian, prostate, bladder or liver cancer cells, low levels of both proteins were observed, which was associated with a worse prognosis in terms of overall survival. Furthermore, high FABP4 levels slowed the development of hepatocellular carcinoma and thus contributed to its reduction in size [69]. This effect is explained by the inhibition of STAT3 phosphorylation via the Ras-p-STAT3 signaling pathway and the inhibition of the Snail protein, an accelerator of the epithelial-mesenchymal transition [69]. However, the increased FABP3 expression in tumors of the stomach, brain, small- and non-small-cell lung cancer seems paradoxical; it has been shown to increase tumor aggressiveness and poorer patient prognosis [70, 71]. Thus far, the molecular mechanism responsible for the oncogenic potential of FABP3 and FABP4 is unknown. Further research is needed to clarify the reasons these proteins promote the neoplastic process. Another subtype of fatty acid binding proteins is FABP5. It is involved in the development of breast, prostate, liver, stomach, and colon cancers. FABP5 has been shown to transport saturated and unsaturated long-chain fatty acids that act as PPARβ/δ ligands [72, 73]. The nature of the supplied fatty acids determines the oncogenic activity, if unsaturated fatty acid or a suppressor action is supplied to PPARβ/δ, in the case of saturated ligands [72]. When the FABP5/PPARβ/δ pathway is stimulated, the transcription of the genes responsible for cell growth and survival increases. Moreover, one of the target genes stimulated by PPARβ/δ is the FABP5 gene, which increases the expression of the transport protein in question [74]. In prostate cancer cells, by supplying long chain fatty acids, FABP5 leads to PPARγ activation [75–78]. Stimulation of these receptors results in increased expression of vascular endothelial growth factor (VEGF), thereby accelerating the angiogenesis process. This mechanism plays a key role in the development of castration-resistant prostate cancer [79–81]. In the case of colon cancer, FABP5 contributes to its development by reducing p21 activity. Overexpression of FABP5 increases the expression of c-MYC, which inhibits the action of a cell cycle inhibitor. After silencing FABP5 activity, a significant reduction in the invasive capacity of colon cancer cells (CRC) is observed [82]. The molecular basis of this interaction is not yet known at the moment. High expression of monoacylglycerol lipase (MAGL), responsible for the production of free fatty acids, is observed in highly malignant colon cancer cells [83, 84]. This fact combined with the physiological role of FABP5, consisting of transporting free fatty acids to the appropriate cell compartments, prompts to seek the answer to the question whether FABP5 and MAGL come into functional interaction with each other, and if so, how does this affect CRC progression. Another FABP isoform that may be involved in colon cancer development is FABP6 [85–87]. It transports bile acids between the epithelial cells of the large intestine, which are transformed into secondary metabolites such as deoxycholic acid (DCA) and lithocholic acid via cellular metabolism. A correlation was demonstrated between increased exposure of intestinal epithelial cells to DCA and an increase in reactive oxygen species inside them which is associated with oxidative stress development and DNA damage [88–90]. This mechanism contributes to the formation of mutations that in the event of a replication of defective cells initiate their malignancy. Due to their detergent properties, bile acids can cause damage to the cell membrane. Compensatory mechanisms lead to an intensified inflammatory response and increased proliferation, which is qualified as early tumor development. Furthermore, bile acids activate the muscarinic M3 receptor [91–93]. Its stimulation induces metalloproteinase activity and the Wnt/B-catenin signaling pathway, which in turn increases the metastatic potential as well as the proliferation and survival of colon cancer cells. Despite the increasing evidence and attempts to explain the mechanisms of FABP action in specific types of cancer, further research is needed to better understand the diagnostic and therapeutic value of these proteins.

### AQPs: aquaporins

**Figure 6:**
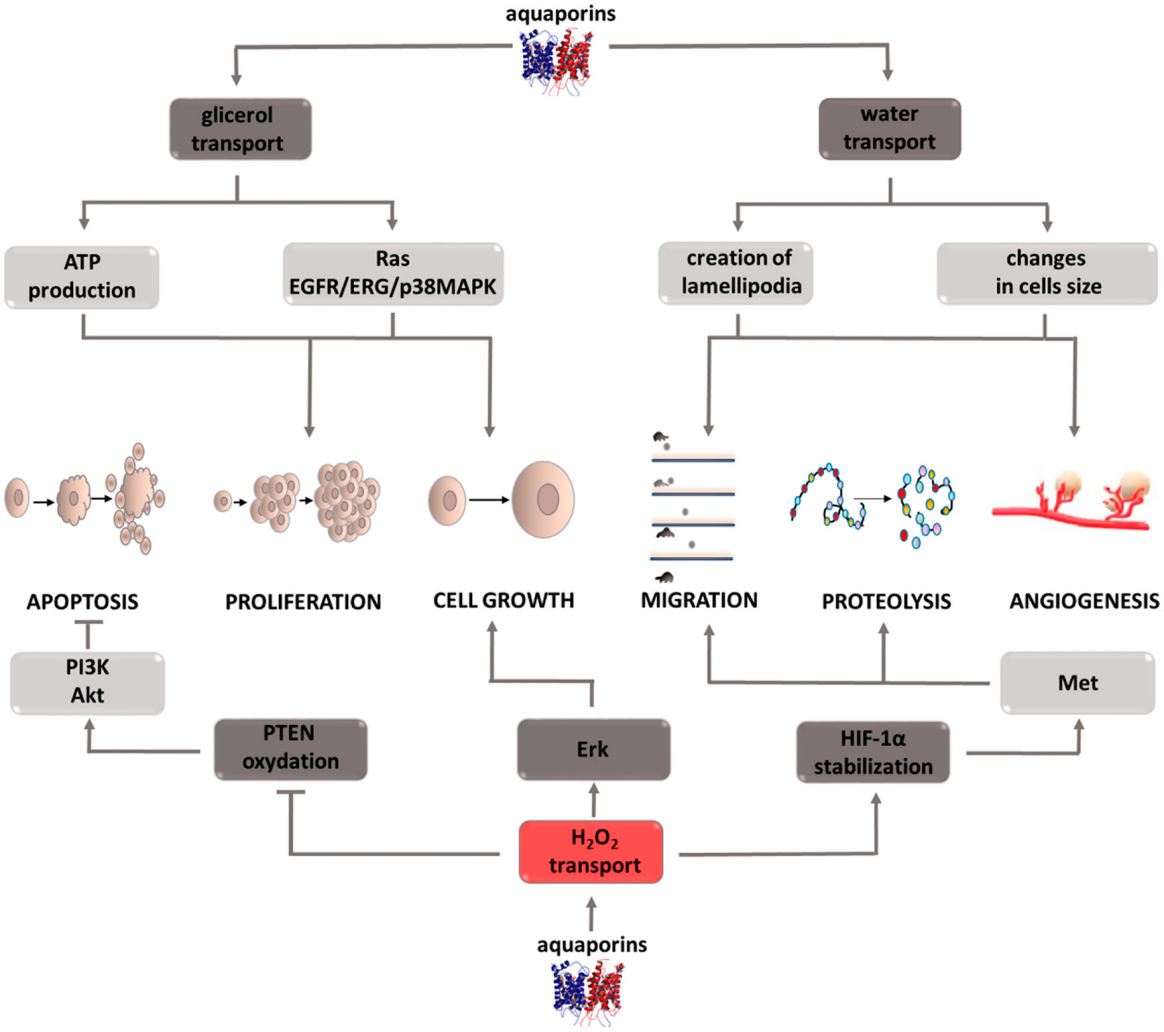
Carcinogenic effect of peroxisome aquaporins (AQPs). The induction of the process is illustrated by arrows, the inhibition by the horizontal line. PTEN: phosphatase and tensin homolog deleted on chromosome ten; PI3K: phosphoinositide 3-kinase; Akt: protein *kinase* B; ERK: extracellular signal-regulated *kinases*; HIF-1α: hypoxia-inducible factor 1-alpha; Met: tyrosine-protein kinase *Met*; ATP: adenosine triphosphate; EGFR: epidermal growth factor receptor; ERG: erythroblast transformation-specific related gene; p38MAPK: *P38* mitogen-activated protein kinases.

Aquaporins (AQPs) are channels responsible for the transcellular transport of water, glycerol, hydrogen peroxide, and other small water-soluble particles. Due to the transported molecules, the family of these 13 proteins can be divided into two subgroups, those that transport only water and those that additionally have the ability to carry glycerol [94]. Regardless of belonging to one of the two described subgroups, aquaporins can participate in H_2_O_2_ transport, which, as it turns out, is of considerable importance in the neoplastic process (Figure 6) [95, 96]. Research conducted on various types of cancer cells, including colon [97], ovarian, [98] brain, [99] lung [100], and pancreatic cancers [101], have shown a correlation between increased aquaporin expression and the level of tumor growth [102]. For this reason, aquaporins have become of interest to researchers exploring processes that play a key role in this pathological process. Tumor cell migration is a stage of disease development in which aquaporins are involved by performing their primary function, i.e., water transport. The migrating cell is characterized by structure polarization and varying build of the leading edge and its end, the so-called uropod. It was observed that aquaporin expression mainly concerns the leading edge, in which the increased influx of water facilitates lamellipodia formation [103, 104]. These structures are the driving force for the wandering cell, and also participate in attaching it to the base in a new site in the body [105]. It is also suspected that aquaporins affect a cell’s ability to migrate through the osmotic engine mechanism [106, 107]. The inflow of water and ions at one end of the cell, and their outflow from the opposite pole can cause motion in and of itself. Moreover, by regulating cell hydration, aquaporins determine its size and shape. The outflow of water causing rapid cell reduction significantly facilitates their squeezing through narrow intercellular spaces and reaching hard to reach places. It is important to note that transporters only support cellular migration, they are not its key perpetrators. Therefore, their inhibition will not completely stop cell movement, but gives hope for its significant reduction. The migration process is also involved in the ability of cells to infiltrate local tissues surrounding a growing tumor [108]. The argument demonstrating the involvement of aquaporins in infiltrate formation is their increased expression in cells that form a tumor’s surface layer, i.e., the one that is capable of infiltration. However, because the mechanism by which these transporters contribute to increased infiltration is unknown, this area remains a field that is requires research. Cell migration pertains to not only cancer cells, it is also a necessary process for effectively rebuilding and creating blood vessels. Because it occurs on the same principle as metastasis, aquaporin activity may be connected with the angiogenesis process. Through their involvement in angiogenesis and cellular migration, these proteins contribute to tumor growth and progression. Cell proliferation, in which aquaporins are directly and indirectly involved, also contributes to tumor growth. The indirect activity involves stimulating signaling pathways such as Ras or EGFR/ERG/p38 MAPK which contribute to the growth and differentiation of propagated cells [109, 110]. The direct activity is associated with the intracellular transport of glycerol. Since glycerol metabolism is associated with ATP production, it seems that it is a source of energy necessary for life processes, especially in the case of rapidly dividing cells [111, 112]. Due to the ability of quick adaptation and utilization of the available nutrients for cancer cells’ own needs, this mechanism seems to be extremely important. An important aspect of the role of aquaporins in intensifying tumor development is their participation in the transport of hydrogen peroxide, which as a representative of reactive oxygen species is involved in proliferation, differentiation, migration, and adaptation to hypoxia or apoptosis. H_2_O_2_ is involved in PTEN oxidation, which in turn activates the PI3K/Akt pathway promoting cancer cell survival [113, 114]. This mechanism illustrates the role of hydrogen peroxide as a secondary messenger. Another example confirming this role of H_2_O_2_ is a study that showed the inhibition of Erk and Akt kinase activity, and thus the weakening of the effects of the EGF pathway after NOX inhibitor administration, the enzyme responsible for reactive oxygen species formation [115]. The role of hydrogen peroxide, and through it aquaporins, in migration and angiogenesis is shown by a study that proved that the targeted cell movement induced by the CXCL12 chemokine requires the presence of both factors [116]. Silencing AQP3 activity inhibits the morphological changes of cells necessary for their movement, while the inhibition of NADPH oxidase, an enzyme responsible for reactive oxygen species formation, prevents actin polymerization on the cell’s leading edge [116, 117]. Stopping these processes significantly limits cell motility and prevents metastasis or blood vessel remodeling. Another mechanism thanks to which H_2_O_2_ promotes metastasis is its effect on HIF1-a under hypoxic conditions. The intracellular increase in hydrogen peroxide concentration has a stabilizing effect on HIF1-a enabling it to translocate into the cell nucleus and to induce transcriptional activity, consisting of Met oncogene stimulation, among others [118–120]. Through it, cells acquire a phenotype that favors features responsible for their invasiveness. The above examples indicate that aquaporins play an important and complex role in intensifying the neoplastic process. For this reason, it can be assumed that therapeutic strategies incorporating inhibitors of these transporters will improve patient outcomes. It should not be forgotten that in many situations aquaporin activity inhibition is only of an auxiliary nature, therefore research is needed to help understand which drug combinations will allow to obtain optimal results. Interest in these structures in terms of oncological treatment is relatively new, which is why there is a need for in-depth research that will help detect possible limitations and potential risks associated with therapy based on aquaporin inhibitors.

### IGF-2: insulin-like growth factor 2

**Figure 7:**
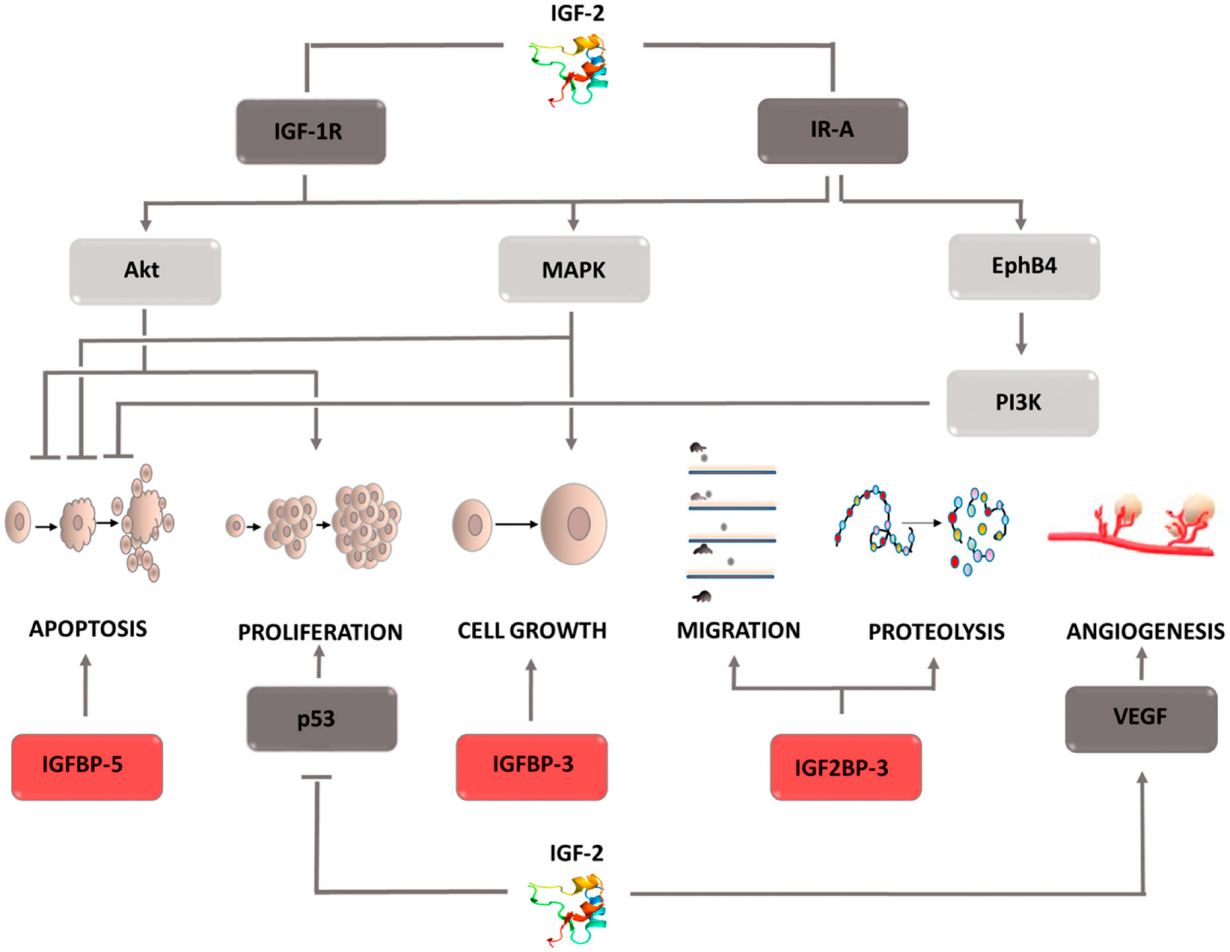
Carcinogenic effect of insulin like factor- 2 (IGF-2). The induction of the process is illustrated by arrows, the inhibition by the horizontal line. IGF-1R: insulin-like growth factor receptor 1 or 2; IR-A: insulin receptor A; VEGF: *vascular endothelial growth factor*; EphB4: Ephrin type-B receptor 4; Akt: protein *kinase B*; MAPK: *mitogen-activated protein kinase*; PI3K: phosphoinositide 3-kinase; IGFBP 3,5: insulin-like growth factor-binding proteins 3 or 5; IGF2BP3: insulin like growth factor-2 mRNA binding proteins 3.

The physiological role of (IGF-2) is to regulate embryonic development. This factor is one of the elements of the complex signaling system, which consists of insulin; two isoforms of its receptor, IR-A, IR-B; insulin-like growth factors, IGF-1, IGF-2; their receptors, IGF-1R, IGF-2R; and two subfamilies of proteins binding this factor, IGFBPs, IGF2BPs. Studies conducted on various types of tumor cells have shown IGF-2 overexpression and increased IGF-1R receptor prevalence [121–123]. This fact is associated with a worse prognosis, shorter survival, and the development of resistance to chemotherapy [124, 125]. Studies conducted to clarify the mechanism of IGF-2 activity have shown that it is necessary to understand the role of the individual components of the insulin/IGF signaling axis. Its elements mutually affect their activity, thereby modulating the final effect. Despite the structural similarities between IGF-1 and IGF-2, their functions differ significantly, indicating greater IGF-2 involvement in the oncogenesis process. Tumor cells produce IGF-2, which has the ability of autocrine and paracrine stimulation of the IGF-1R receptor [126, 127]. Furthermore, they secrete immature IGF-2, i.e., large IGF-2, which has the ability to stimulate the same receptors as IGF-2 and is not susceptible to degradation when combined with the specific IGF-2R receptor. Thus, tumor cells gain the ability to stimulate the signals involved in the survival processes even in the presence of a suppressor protein. One of the main effects of IGF-2 is IGF-1R receptor stimulation, leading to autophosphorylation of the β-subunit, recruitment of the insulin receptor substrate (IRS), and activation of the Akt, MAPK signaling pathways (Figure 7) [128]. IGF-2 stimulation of the IR-A receptor has a similar effect. The active Akt kinase inactivates the proapoptotic BAD proteins, thus inhibiting cell death. It also enhances the synthesis of the ribosomal proteins necessary for the mitosis process by activating the mTOR pathway and affects the transfer of glucose transporters (GLUT) to the cell surface. As a result, the supply of energy to the dividing cells is increased. Activation of the MAPK pathway, in turn, causes changes in the expression of the protein genes necessary for cell growth. Both pathways serve to support cell life and proliferation. Interesting conclusions were provided by the discovery of Panadini *et al.* who showed that IGF-2 inhibits the ubiquitination of EphB4 receptor tyrosine kinase by autocrine stimulation of insulin receptor A isoform. This effect is exerted regardless of IGF-1R receptor stimulation and results in PI3K signaling pathway activation by EphB4 [129]. This explains why therapy based on IGF-1R inhibitors does not bring the desired results, and tumor cells do not lose the ability to develop, proliferate, and metastasize [130]. Understanding the mechanism of drug resistance and the role of IGF-2 inhibitors in its formation may contribute to the development of a new therapeutic strategy in the fight against cancer. It is currently assumed that the failure of IGF-1R inhibitor therapy is only associated with IR-A receptor stimulation. However, there is no data that would explain whether it can also be caused by the ability to stimulate the hybrid receptor IR/IGF1R by IGF-2. In addition to its effect on signaling cascades, IGF-2 has the ability to inhibit p53 activity, which obviously contributes to the proliferation of cells containing defective genetic material. IGF2 is not only involved in cell proliferation and viability, it also participates in the angiogenesis process by affecting stem cell differentiation toward endothelial cells. Moreover, hypoxia-induced factor enhances IGF-2 expression in conditions of insufficient oxygen supply, which then activates VEGF expression. Thus, IGF-2 contributes to the creation of the optimal tumor microenvironment, which provides it with the transport of factors necessary for growth. As mentioned earlier, the whole system also includes the receptor IGF-2R, which when combined with the ligand leads to IGF-2 degradation. Furthermore, it can release its extracellular domain into the bloodstream, which binds circulating IGF-2, thereby inhibiting its activity. Therefore, this receptor is considered a tumor suppressor factor. The amount of IGF-2 circulating in the circulatory system is regulated by specific proteins called insulin-like growth factor-binding proteins (IGFBPs). It is a family of 6 forms of proteins that modulate IGF-2 activity by blocking its binding to the receptor. The expression of individual IGFBPs is tissue-specific, and the induced effects differ. For this reason, it is impossible to assign a specific role in the cancer process to the entire family. For example, IGFBP-5 found in mammary glands inhibits differentiation and enhances the apoptosis process in breast cancer cells. IGFBP-3 in esophageal cancer cells acts as an antioxidant, thereby enhancing tumor cell growth in an IGF-2-independent mechanism [131, 132]. It also affects epithelial-mesenchymal transition, intensifying the formation of cancer stem cells. The second family of proteins that affect IGF-2 are IGF-2 mRNA binding proteins (IGF2BPs), which has three components. Their role in the cancer process is better understood. An increase in IGF2BP3 expression is associated with increased cell invasiveness and greater dynamics of the tumor process IGF2BP2, in turn, stabilizes IGF-2 mRNA, which increases its expression contributing to tumor development [133–135]. These examples indicate how extensive and important a role insulin-like growth factor 2 plays in the cancer process. For this reason, it is fully justified to conduct research enabling the development of pharmacotherapy based on IGF-2 inhibitors. It should not be forgotten that it is a component of a complex signaling system that also regulates physiological processes. Thus, when designing treatment, the dependencies connecting individual components and potential threats resulting from inhibiting the activity of one of the pillars should be taken into account.

### Exosomes

**Figure 8:**
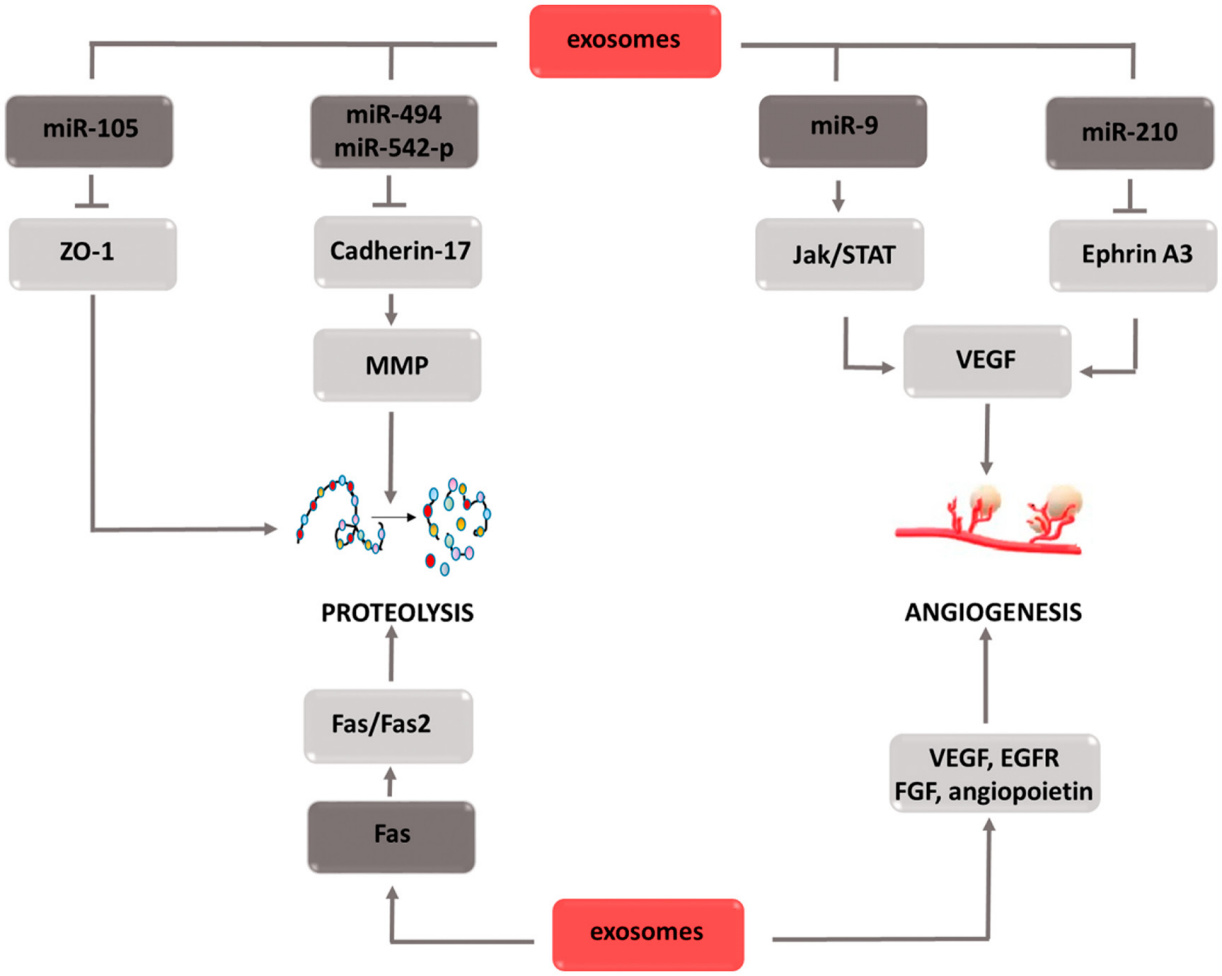
Carcinogenic effect of exosomes. The induction of the process is illustrated by arrows, the inhibition by the horizontal line. miR: micro ribonucleic acid; ZO-1: zonula occludens-1; MMPs: *metalloproteinases*; JAK: Janus-activated *kinases*; STAT: signal transducer and activator of transcription; VEGF: vascular endothelial growth factor; FGF: fibroblast growth factor; EGFR: epidermal growth factor receptor.

Exosomes are small membrane vesicles secreted by both normal and cancer cells. Acting in an autocrine or paracrine manner, they participate in intercellular communication processes. The basis of this phenomenon is two separate mechanisms, i.e., the delivery of proteins that affect signaling cascades to target cells, or the transport of various micro RNAs (miRNAs) that modulate the expression of individual genes. By affecting tumor transformation, angiogenesis and metastasis, exosomes are one of the factors that accelerate cancer progression (Figure 8). The effect on the transformation of normal cells into malignant cells is mediated by various types of miRNAs that activate oncogenes and inhibit the expression of suppressor genes [136]. Moreover, exosomal miRNAs stimulate receptors in the TLR (Toll-like receptors) family, which leads to a chronic inflammatory response, one of the factors intensifying tumor transformation [137, 138]. In addition, the release of proinflammatory cytokines caused by TLR stimulation leads to neutrophil recruitment in the inflammation area. As a source of reactive oxygen species, neutrophils can destabilize and damage epithelial cell genetic material, thus contributing to their mutation and malignancy [139]. Due to the ability to create neutrophil extracellular traps (NETs), they can trap circulating tumor cells (CTCs) and then transport them to areas distant from the primary tumor focus [140–142]. Furthermore, by secreting factors of vascular endothelial activation, neutrophils increase its adhesion, which facilitates the adhesion of the transported CTCs [143–145]. Thus, the formation of metastasis in a region distant from the primary tumor is significantly simplified. The described mechanism combining the effect of exosomes with the effects caused by neutrophils is not the only one by which cell vesicles are involved in the particular stages of tumor development. By directly supplying factors such as VEGF [146, 147] EGFR [148] fibroblast growth factor (FGF) [149] or angiopoietin [150] they contribute to new blood vessels development that ensures the optimal growth environment for the dividing cells. The indirect pro-angiogenic activity is the transport of miR-210 through exosomes secreted by tumor cells [151, 152]. Under hypoxic conditions, miR-210 has the ability to inhibit ephrin-A3 receptor tyrosine kinase expression [153–155]. As a result of this interaction, increased secretion of VEGF occurs, which stimulates the formation of new blood vessels [156, 157]. To stimulate VEGF gene expression, exosomal miR-9 contributes indirectly, which activates the JAK-STAT signaling pathway [158–160]. Thus, STAT3 factor, a direct transcription activator of the VEGF gene, is phosphorylated [161]. The aforementioned neutrophil extracellular traps (NETs) contribute to metastasis formation. Another mechanism involved in this stage of carcinogenesis is the weakening of tight junction intercellular connections by regulating the ZO-1 (zonula occludens-1) gene. Exosomal miR-105 inhibits the expression of this gene, which causes connection loosening and the destruction of the barrier formed by epithelial cells [162–164]. Thus, cancer cells gain access to the blood vessel lumen, which creates the possibility of moving to remote areas of the body. The down regulation of cadherin-17 expression induced by exosomal miR-494 and miR-542-p transport also favors migration [165, 166]. A decrease in the concentration of this protein leads to an increase in metalloproteinase concentration, thereby increasing their activity. Metalloproteinase activity also increases via the Fas/Fas2 signaling pathway [167–169]. When transporting the Fas ligand to the target cell, exosomes contribute to the activation of the Fas/Fas2 pathway, and thus increase the activity of the proteins responsible for the reorganization of the extracellular matrix [170, 171]. The second effect induced by active metalloproteinases is growth factor release. Thanks to these two mechanisms, enzymes not only facilitate the movement of cancer cells, but also have a positive effect on the angiogenesis process. The described relationships indicate that exosomes are involved in the complex mechanisms leading to the development of the individual stages of carcinogenesis. Therefore, the use of inhibitors of their activity would enable comprehensive inhibition of the tumor progression process. For this reason, these components appear to be an attractive therapeutic target that, if included in the treatment strategy, could bring measurable benefits.

### Carcinogenesis modulators: from basic research to clinical practice

Existing data indicate that all factors considered in this papier represent promising and challenging therapeutic targets. That provides the basis for developing new anticancer drugs or testing known agents that are already used in clinical practice to repurpose their indication. This part of the manuscript provides an overview of the studies that reveal the connection between preclinical knowledge (both *in vitro* and *in vivo*) and its practical use.

The AChRs inhibitors intended to provide treatment for irritable-bowel syndrome or overactive bladder are currently being tested in different cancer types. An *in vitro* study showed that M1 muscarinic receptor antagonist dicyclomine and/or M3 muscarinic receptor antagonis, derifenacine reduced viability and increased apoptosis of chemoresistant lines of non-small cell lung cancer (NSCLC) and prostate cancer (PCa) [172]. In turn, another M3 muscarinic receptor antagonist, aclidinium bromide inhibits growth and limits the metastatic potential of A549-human lung cancer cells [173]. Witayateeraporn *et al.* also showed the potential benefits of anticholinergic treatment in cancer therapy. They tested four nAChR inhibitors (QND7, PPRD10, PPRD11, and PPRD12) on two cell lines of NSCLC (H460, A549) and indicated that QND7 showed the most significant cytotoxic effect, induction of apoptosis and lowering cell migration. The authors suggested mTOR/Akt pathway involvement in the antitumor effect of QND7 [174]. Promising outcomes are also provided by the studies conducted on the Swedish population. An inversely proportional relationship was established between exposition on antimuscarinic treatment, using compounds such as oxybutynin, solifenacin, darifenacin, fesoterodine, or tolterodine and the risk of colon or lung cancer [175].

Rosiglitazon, a widely used PPARg agonists, has been tested in a number of oncological studies. An *in vitro* study on two breast cancer cell lines (A2780, SKOV3) revealed severe apoptosis and tumor growth inhibition in mouse xenografts after combined therapy of rosiglitazone and olaparib [176]. Rosiglitazone also decreased proliferation and tumor size in human squamous cell carcinoma (SCC) xenografts [177]. In turn, Lau *et al.* demonstrated the synergistic impact of rosiglitazone on 5-fluorouracil-induced apoptosis of two colorectal cancer cell lines (HCT-116, HT-29). The effect of this combination was significantly stronger compared to 5-FU in monotherapy [178]. Another, PPARg agonist, trioglitazone significantly suppressed the growth of human oral squamous cell carcinoma cell lines (HSC-4, Ca9-22) by inducing cell cycle arrest [179]. The efficacy of rosiglitazone and pioglitazone, has also been evaluated on bladder cancer cells. Both drugs induced cell cycle arrest and decreased proliferation of two tested cell lines Umuc-3 and 5637. Pioglitazone, also appeared to be effective in relation to melanoma. The results of an animal study show that pioglitazone inhibits subcutaneous tumor growth in a mouse xenograft model of murine melanoma B16F10 cells [180]. Moreover pioglitazone in combination with rofecoxib and trofosfamide were also active and well tolerated in patients with chemorefractory melanoma and soft tissue sarcoma [181].

Therapy with Btk inhibitors and its potential benefits are now in development. Combination therapy of ibrutinib (Btk inhibitor) and 5-FU resulted in an increase in the cytotoxicity of 5-FU resistant human colorectal cancer. Similarly, other Btk inhibitors (AVL-292, RN486, CGI1746, ONO-40590) used in the combination with 5-FU also inhibited the growth of these cells via enhancement of apoptosis. Moreover, a cytotoxic response was obtained on patient-derived 3D organoids after the administration of 5-FU with ibrutinib or AVL-292 [182]. In the mouse xenograft model resensitization to 5-FU in HCT116p53KO cells in the presence of ibrutinib was observed [182]. In turn adding erythropoietin to LFM-A13 (Btk inhibitor) significantly intensified the anticancer action of LFM-A13 in colon cancer xenografts [183]. BTK inhibitors were generally evaluated on hematological malignancies. Ibrutinib is currently approved for use in patients with chronic lymphocytic leukemia (CLL) or mantle cell lymphoma (MCL) and has an established safety record from clinical trials in these patient populations. An increasing body of experimental and clinical data from recent years supports the major role of Btk not only in B cell malignancies [184] but also in other solid tumors, including breast [185], ovarian [186], and prostate cancer [187].

The described role of FABPs depicts them as agents that play a double role in carcinogenesis. Therefore, selectivity for each isoform may prove to be a key determinant of the pro/antitumor role of potential therapeutic drugs. The literature data indicate berberine as the inhibitor of FABPs expression. Human gastric cancer cells MGC803 and MGC803 xenografts were exposed to its action. Both *in vitro* and *in vivo* results indicate increased apoptosis and inhibition of proliferation. Another tested FABPs inhibitor BMS309403 also exerted antiproliferative activity and limited tumor growth. Simultaneous use of both compounds generated stronger growth inhibition compared to administered alone [188]. Hsiao *et al.* observed FABP5 mRNA suppression and HT-29 cell migration inhibition after pterostilbene treatment. They emphasized that pterostilbene, as a FABP5 inhibitor, can serve to mitigate adiposity-induced metastasis in CRC [189]. Additionally a clinical trial to assess megestrol acetate with or without pterostilbene in treating patients with endometrial cancer undergoing hysterectomy is ongoing.

As mentioned above, AQPs inhibition could be helpful in supporting anticancer therapy, especially in terms of metastasis. A recent study demonstrated furans as a new class of AQP1 ion channel inhibitors that block slow cancer cell migration and invasion. An *in vitro* study on cell cultures with different expressions of AQP1 showed that 5HMF (5-hydroxymethyl furfural) significantly reduced the migration of cells with high AQP1 expression (colon cancer cell HT-29, breast cancer cell MDA-MB-231) without causing changes in cells lacking AQP1 expression (colon cancer cell SW480) [190]. Similar results were obtained in a trial that evaluated the effect exerted by AqB011 and bacopaside II in monotherapy or in combination. Both compounds were identified as AQP1 inhibitors with two different mechanisms of action. AqB011blocks the ion pore of AQP1, whereas bacopaside II targets the water pore, which prevents a water flux.

The attenuation of HT-29 cell migration in monotherapy has been demonstrated as well as a significant improvement of this process in combined therapy. Moreover, a decreased invasiveness of cells was observed after bacopaside II treatment [191]. A similar effect was demonstrated in colon cancer cells (HCT116, SW480) after AQP-5 knock-down with specific shRNA [192]. The anti-cancer activity of the extracts bacopaside I and bacoside A in mouse models of melanoma, sarcoma and Ehrlich ascites carcinoma was also shown. Existing data concerning other forms of AQPS indicated that auphen (AQP3 inhibitor) decreased expression of AQP3 aquaporins and suppressed the development and tumor growth of xenografts of the HCC cell line [193].

The next factor considered in the manuscript, that can be a potential antitumor target is IGF-2. Xentuzumab (IGF ligand-neutralizing antibody) was examined in monotherapy or in combination with enzalutamide on five prostate cancer cell lines (VCaP, DuCaP, MDA PCa 2b, LNCaP, and PC-3). In both therapeutic options cell viability was significantly decreased in all tested cell lines. Moreover xentuzumab + enzalutamide inhibited the growth of castration-resistant LuCaP 96CR PDX with acquired resistance to enzalutamide, and improved survival *in vivo* [194]. Nevertheless the addition of xentuzumab to enzalutamide did not prolong progression-free survival in patients with metastatic castration-resistant prostate cancer compared with enzalutamide alone [195].

Exosomes based therapy can be focused on a few mechanisms of action. Potentially useful molecules can act as the inhibitors of endocytosis (i.e., heparin, cytochalasin D, methyl-β-cyclodextrin), limit cell membrane fusion or limit their biogenesis and release (in this approach Rab proteins, as the biogenesis modulators can be targeted) [196]. Because of the role of exosomes as the communicators between cells, they can be used as well, to transport antitumor molecules. For the internalization exosomes bind with HSPG (Heparan sulfate proteoglyca), which also binds with heparin [197]. Interaction between heparin an HSPG concurs with exosomes binding, thus disturbs theirs uptake. From this reason heparin is tested as a potential anticancer agent. Pretreatment oral squamous carcinoma cells (OSCC) with heparin allowed for temporary inhibition of exosomes uptake. In turn multiply heparin administration significantly reduced cells’ proliferation, migration and invasiveness. This observation has been repeated *in vivo*, on xenograft nude mice model, in which constant heparin infusion decreased tumor growth [198]. Anticancer effect of heparin was observed also in the urothelial cells treated with muscle-invasive bladder cancer- derived exosomes (MIBC). Incubation of primary urothelial cells with MIBC exosomes increased theirs migration, what was reversed after heparin pretreatment [199]. As mentioned before, molecules which can be essential in exomes release are Rab proteins, which control exocytosis. Especially, Rab11, 27a/b, 35 are involved in this process [200]. It was shown that in the prostate cancer cells tipifarnib (farnesyltransferase inhibitor) decreased level of Rab27a, with following inhibition of exosomes’ release. Similar effect was gained on the same cell line after administration of neticonazole, climbazole and ketoconazole [201]. The latter is already use as a second-line strategy in castration-resistant prostate cancer [202]. Neticonazole lowered exosomes serum level induces apoptosis and inhibits tumor growth in colorectal cancer xenograft mouse model [203]. Tipifarnib as a monotherapy of metastatic head and neck squamous cell carcinoma (HNSCC) bearing HRAS mutation in the II phase of clinical trial showed durable response and lack of progression in most of the patients [204].

## CONCLUSIONS

The pathogenesis of cancer is extremely complex and depends on many factors. Often, one element affects several stages of carcinogenesis, and individual stages are regulated by many stimuli. This may be an explanation for the difficulties in conducting effective cancer therapy. Pharmacological inhibition of one way of development in a way forces cancer cells to incorporate other mechanisms that will enable their survival. On the other hand, the multifaceted action of the described causative agents gives hope that the use of targeted substances can contribute to the inhibition of key life processes and stop tumor growth. In-depth knowledge of the role of individual carcinogenesis modulators creates the basis for the development of new drugs that may prove to be an effective weapon in the fight against cancer. Moreover, this knowledge may enable the right selection of drug binding sites in the development of combination therapy and contribute to safer treatment with fewer side effects. The constantly growing dynamics of cancer spread indicates for a need to also intensify research in the aspect of factors modulating the carcinogenesis process. Full understanding of the mechanisms responsible for carcinogenesis is an extremely difficult challenge, but it is necessary in an effective fight against cancer.
